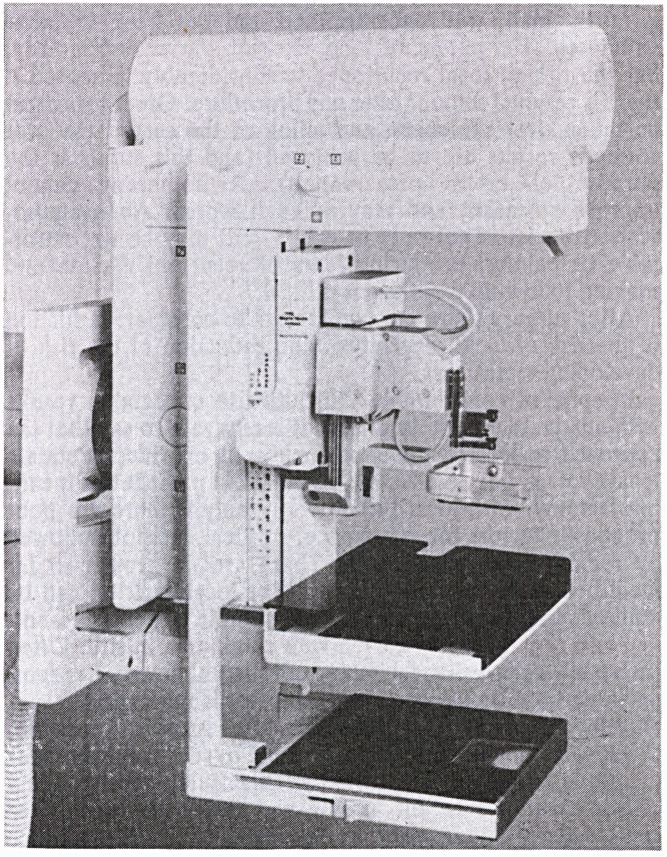# Technical Data from Philips

**Published:** 1989-05

**Authors:** 


### Technical Matters

### THE CYTOGUIDE—A STEREOTACTIC BIOPSY SYSTEM From Philips Medical Systems.

Mammography has generally been acknowledged to be the best method for diagnosing breast cancer during the occult stage. As breast tissue changes may, in some cases, be as small as a few millimeters and therefore impossible to localize by means of palpation, it is important that any biopsy or localization performed is both diagnostically precise and geometrically accurate. Disadvantages of the fine-needle biopsy system are those combined with the risk of false-negative examination results. The risk should, however, decrease if higher precision with the biopsy needle is achieved and lead to a diminishing need for excision surgery.

The use of a stereotactic biopsy instrument enables a more accurate and precise biopsy needle incision to be performed, as it is possible for the point of insertion of the biopsy needle, the depth of insertion and direction to be defined and controlled precisely with the aid of a biopsy guide.

Accurate localization on non-palpable problematic sites can be achieved with the sterotactic biopsy unit, the CYTOGUIDE, which has been adapted for use with the Philips Mammo Diagnost U/M.

The Cytoguide consists of a lightweight biopsy unit, a desk top operator console and a hand-held operating panel. The biopsy unit, which is normally stored on a lightweight wall bracket, slides easily on to the object table and is locked into place via solenoid brakes. The biopsy cone which incorporates a near focus sliding diaphragm with reference mark is inserted into the cone holder. The tube arm can be positioned between $\pm 90°$ to facilitate the biopsy procedure.

A self-test programme ensures that the dual needle guidance system starts from a zero position for each biopsy procedure.

For a stereotactic exposure, the film cassette in the cassette holder and the diaphragm slide are moved to the far right position and the tube arm tilted to $+20°$.

A subsequent exposure is made with the cassette and diaphragm slide in the far left position and the tube arm tilted to $-20°$.

An additional Amplimat ionization measuring chamber is incorporated in the Cytoguide biopsy unit, it is located directly behind the object ensuring consistent film density.

The film, once developed, is placed over the light window on the operators console, displaying the stereotactic views of the region of interest and the superimposed reference markings. The co-ordinates of the region of interest are now determined.

Using the cursor and following the instructions in the LCD panel of the control desk, the co-ordinates are entered into the computer via the ENTER key. Once the required needle length has been chosen, the dual needle guidance system automatically moves to the region of interest. The height of the dual needle guidance system is set, so that when the tip of the biopsy needle is subsequently inserted to its maximum depth, it is accurately positioned within the region of interest.

The on-line evaluation system provides accurate needle positioning, with an overall accuracy of $\pm 0.5$ mm. No time consuming manual adjustments are necessary.

The safety switch is then pushed back, locking all movements and releasing the needle insertion guide. The lower needle guide is placed as close as possible to the breast tissue so that needle deflection during tissue penetration is minimal. Sterilized guide bushes in the upper and lower needle guides again ensure minimal needle movement as well as hygiene between examinations.

If sampling of cells from a larger region is required, then further insertions can be carried out by changing the target co-ordinates in steps of $\pm 0.1$ mm in either the X, Y or Z directions using the hand-held panel.

The Cytoguide minimizes the disadvantages associated with fine needle biopsy procedures, since it provides a method for performing both diagnostically precise and geometrically accurate biopsies or localizations. This accuracy of $\pm 0.1$ mm ($\pm 0.5$ mm overall) together with the lightweight design and simple user instructions enable the stereotactic biopsy procedure to be performed both quickly and efficiently to the patient's advantage.

Women are better informed than they used to be, and may have personal experience of the disease in friends or family members. They may have erroneous beliefs or fears about their own illness but are more likely to ask about different approaches to treatment. Paradoxically, the appreciation of the risk of relapse can make it easier for the doctor to discuss adjuvant treatment.

The most recent source of anxiety is breast screening. Women worry about not being screened and whether it will make any difference to them. Doctors do not know whether early cases will do best with conventional treatment or whether new approaches will be necessary. Women showing false positive results will need strong reassurance, and some of them will undoubtedly develop cancer eventually.

We already have plenty to do when it comes to treating breast cancer, as I hope I have shown. Earlier diagnosis will not reduce our workload; in the short term it will increase it. Treatment of metastatic disease is unlikely to change greatly but patients with early disease will be subjected to increased scrutiny. 'Who will develop metastases?' If we can answer this question we can intensify our treatment in this group and spare the rest unnecessary side effects. The answer is still a long way off.

## Figures and Tables

**Figure f1:**